# Monitoring and redirecting virus evolution

**DOI:** 10.1371/journal.ppat.1006979

**Published:** 2018-06-07

**Authors:** Gonzalo Moratorio, Marco Vignuzzi

**Affiliations:** Viral Populations and Pathogenesis Unit, Institut Pasteur, CNRS UMR 3569, Paris, France; Mount Sinai School of Medicine, UNITED STATES

## Introduction

“Nothing in biology makes sense except in the light of evolution."—Theodosius Dobzhansky

Viral pandemics can kill more people than war. From biblical plagues to modern-day outbreaks, the ensuing devastation is in part driven by the extreme genetic diversity of these pathogens, which allows them to rapidly evolve to escape immunity, jump to new species, and enter new ecological niches. The quote above, penned by a central figure who helped shape the modern-day synthesis of evolutionary science, is oft encountered today as a reminder that evolution remains relevant to all biology and certainly to infectious diseases. Indeed, throughout our history, whether deliberately or unintentionally, we humans have tried to control this very force responsible for our existence. And more recently, as microbiologists, advances in computer science and sequencing technology have moved our field to the next level. We can now follow, almost in real time, the molecular epidemiology of emerging outbreaks, allowing us to directly test field observations in the lab. What methods are available to follow viral evolution? Is it possible to predict virus evolution? Can we use this knowledge to drive deadly pathogens towards evolution's dead end, to extinction? These questions will guide the reader through this review.

## How to first characterize the emergence of viral outbreaks? By sequencing and phylogenetics

**Fig 1:**
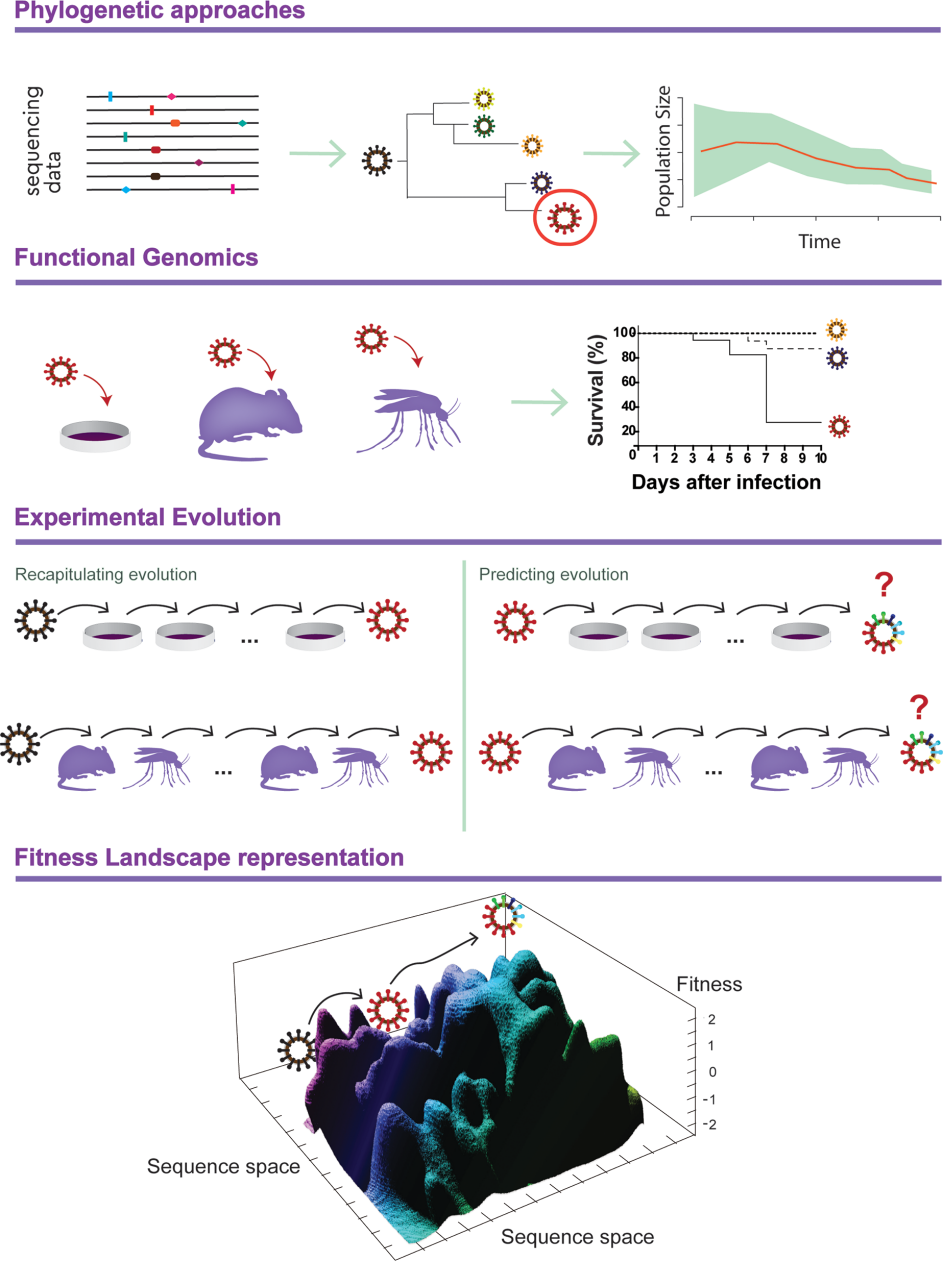
Monitoring virus evolution. Top panel: Phylogenetic approaches are first used to characterize a viral outbreak. From left to right: Sequencing data identifies a new viral genotype by phylogenetics. This new strain is represented in red in the phylogenetic tree. By combining these data with sampling dates, a Bayesian skyline plot reveals the demographic history of the epidemic. Middle panels: First, by functional genomics, this new variant is tested in vitro and in vivo. Survival curves show the phenotype of this new strain. Second, experimental evolution is performed to a) recapitulate the evolution observed in nature using ancestral genotypes and b) predict the next mutations likely to emerge, using as a starting point the newly identified variant (in red). Bottom panel: Implementation of genotype–phenotype maps would help monitor evolution and potentially predict future trajectories towards, or away from, virulence.

We employ phylogenetic approaches to study the evolutionary history and relationships of organisms. Heritable traits, such as genetic sequences, are represented in phylogenetic trees (Fig 1, top panel, center), which portray relatedness between organisms by branches and in which the length of branching lines represent evolutionary time. Phylogenetics provides an integrative perspective of the identity, classification, ecology, and evolutionary history of viral strains [1]. Usually, it involves mathematical models implementing mainly three kinds of methods: a) parsimony, a criterion of ‘frugality’, based on favouring the simplest explanation or fit of the data, in which the best phylogenetic tree is one that requires the fewest evolutionary changes; b) maximum likelihood (ML), based on inferring probability distributions such that the more likely a viral strain (sequence) falls within a cluster of a given tree, the more that tree is validated; and c) Bayesian inference, which relies on ML but integrates the probability of hypothesis according to previous data (prior probability) to define the best tree. All aforementioned methods are used to analyze genetic relationships among viral strains [2]. Bayesian approximations favoured the integration of sophisticated sampling methods such as Markov chain Monte Carlo (MCMC) algorithms, revolutionizing phylogenetics through the incorporation of complex models of evolution and the estimation of parameters such as substitution rates, divergence times, and other population genetics patterns [3]. In addition, the number of infections over time, inferred from Bayesian skyline plots, allows the reconstruction of the demographic history of a pathogen during epidemics (Fig 1, top panel, right) [4–6]. These advances were significantly boosted by the increasingly abundant genomic data from next-generation sequencing techniques, which are moving from core facilities to benchtop and even to field-friendly platforms. Altogether, this workflow makes near-real-time molecular epidemiology a feasible endeavour. These approaches were used to reconstruct the emergence and spread of recent Zika virus epidemics in the Americas, identifying the timing and sources of introductions in different geographical regions [7]. Similarly, real-time molecular epidemiology of the recent Ebola outbreak in West Africa revealed considerable disconnection between transmission clusters and independently evolved lineages [8,9]. Phylogenetics thus builds a clearer picture of virus outbreaks by identifying evolutionary and transmission patterns.

## How to test new findings made from field samples? By comparing the phenotype of new mutations with the original strain

After phylogenetic characterization, newly identified mutations or viral strains can be tested in the lab in what is known as functional genomics. These approaches, which attempt to mimic what is happening in the wild, reveal how new mutations impact viral phenotype. There are many examples of newly identified mutations associated with antiviral resistance, higher pathogenicity, increased transmission, and cross-species transmission that were confirmed by these means [10–13]. For instance, a single mutation (A188V) in a nonstructural protein (NS1) of Zika virus was first identified by phylogenetics and then shown to increase infectivity in laboratory mosquitoes [14]. The E1-A226V mutation in chikungunya virus that contributed to its jump from *Aedes aegypti* to *A*. *albopictus* mosquitoes was also identified by phylogenetics and later confirmed by functional genomics [15,16]. Sequence analysis of viruses from an outbreak of highly pathogenic H7N7 avian flu in the Netherlands identified 15 amino acid differences between a fatal case and other isolates [17]. Functional genomic studies in mice later identified a previously reported host range mutation (E627K) in the PB2 gene [18,19] that increased viral pathogenesis in vivo [20]. On the other hand, new phenotypes can also result from a constellation of mutations at the viral population level. Although these observations have been only seen in tissue culture, they are still significant and could be the starting point to test it in vivo [21,22]. However, to better achieve mechanistic understandings, we still need to focus on a few mutations and their immediate effect on fitness. Thus, once a mutation of interest is identified and its impact on phenotype confirmed, one can set out to determine under what conditions this emergence event occurred. To do so, experimental evolution can be performed in vitro or in vivo.

## How to reproduce in the lab the genetic and phenotypic changes that have occurred in nature? By experimental evolution

Experimental evolution tries to recreate the evolutionary dynamics surrounding the emergence of a new adaptive mutation in a controlled environment and within a specific time frame. By testing different host environments or viral genotypes, one tries to determine why some mutations occur and others do not. In our own lab, we showed that the chikungunya virus E1-A226V adaptive mutation, mentioned above, arises readily in *A*. *albopictus* mosquitoes in only seven days, and we identified potentially newly emerging mutations in the same gene [23]. In studying the emergence of canine parvovirus by experimental evolution in vitro, Allison and colleagues recapitulated several mutations observed in natural isolates that were specific to host and virus genetic background and confirmed their fitness advantages over the parental virus [24]. Experimentation can be taken one step further, to identify the determinants that can either increase transmissibility, as was performed for H5N1 influenza virus [25], or decrease virulence, which has been used in live-vaccine attenuation for decades [26]. These approaches thus require special consideration before, during, and after the experiments, since experimental evolution in specialized environments could lead to gain-of-function mutations. While powerful in approach, experimental evolution does have limitations, including stochasticity, population size, limited environments, and resources, and although we cannot be sure these mutations will appear in nature, their identification does give us an advantage as chess masters by providing a short list of ‘next moves’ available to a virus.

## How to determine whether new viral genotypes can lead to more pathogenic phenotypes? By implementing fitness landscapes

The fitness landscape is a helpful concept to metaphorically visualize evolution and to uncover how genotype relates to phenotype, a central goal in evolutionary biology [27]. We can consider fitness landscapes as a sort of Global Positioning System (GPS) device, whereby knowing the coordinates of an individual (genotype) allows us to predict how well an organism is positioned in its current environment (fitness, phenotype) and where it is likely to go next. Usually, fitness is represented by the “height” of the landscape, which is grounded on a plane that denotes the genotypic space: Peaks are good places to be (higher fitness), whereas valleys are not (lower fitness). This metaphor has been inching towards reality thanks to multidisciplinary approaches that combine computer science and applied maths with high throughput, highly quantitative biological data [28]. For instance, more than 70,000 HIV-1 samples were used to measure the reverse transcriptase and protease fitness in the presence and absence of different antiviral drugs. Fitness measurements were then coupled with sequencing data and fitted to different mathematical models. This work provided a predictive model based on a biologically relevant fitness landscape, which predicted more than 50% of the fitness values obtained [29]. On the other hand, the process of acquiring beneficial mutations could thus be visualized within the fitness landscape. Such adaptive walks could be further investigated by reconstructing ancestral genotypes and submitting them to experimental evolution. Indeed, this was the case in recapitulating the evolutionary pathway to virulence of the oral polio vaccine [30]. Knowing the ancestral virus and sequences from vaccine-derived poliovirus infections from different outbreaks, Stern and colleagues combined mathematical models and experimental evolution to retrace each mutation and recombination back to the virulent form [30]. Although the trade-off between fitness and pathogenesis is not always reciprocal, a better understanding of genotype–phenotype maps would help monitor evolution and potentially predict future trajectories towards virulence.

## Can we modify an RNA virus’s evolution to our own benefit?

As previously mentioned, the valleys in fitness landscapes are detrimental regions, corresponding to mutations on nonviable or low-fitness genotypes. In principle, we could use this knowledge to redirect virus evolution towards a more dismal future by increasing the likelihood of acquiring such detrimental mutations. Among substitutions, stop codons are possibly the worst mutation an organism could acquire. This idea was directly tested in our recent work in which two very different RNA viruses, influenza A and Coxsackie B3, were genetically engineered to generate more stop codons after replication occurs. These viruses were re-coded in serine and leucine codons by synonymous mutations that placed them only one mutation away from stop codons. Both engineered viruses generated more stop mutations, in vitro and in vivo, accompanied by significant losses in viral fitness and attenuation in mouse models [31]. These findings were in agreement with previous studies in which the ability of RNA viruses to buffer mutation (known as genetic robustness) was reduced. Importantly, these works show that the same error rate will differently impact a genome depending on the codons it carries, which can lead to distinct amino acid changes. Thus, the position that a virus occupies in genotypic space defines not only its mutational robustness but also its mutant spectrum, the evolutionary trajectories available to it, and ultimately its evolvability [32]. The endeavours described are still in their infancy, but the goal to characterize the local sequence and fitness landscape of a virus, to monitor and potentially predict its immediate future, and to evaluate how antiviral approaches might alter these trajectories seems attainable. The further integration of mathematical modelling, bioinformatics, and experimental evolution will booster our response to viral outbreaks.
